# Beta Blockade Prevents Cardiac Morphological and Molecular Remodelling in Experimental Uremia

**DOI:** 10.3390/ijms25010373

**Published:** 2023-12-27

**Authors:** Shanmugakumar Chinnappa, Azhar Maqbool, Hema Viswambharan, Andrew Mooney, Laura Denby, Mark Drinkhill

**Affiliations:** 1 Department of Nephrology, Doncaster and Bassetlaw Teaching Hospitals NHS Trust, Doncaster DN2 5LT, UK; 2 Leeds Institute of Cardiovascular and Metabolic Medicine (LICAMM), University of Leeds, Leeds LS2 9JT, UK; a.maqbool@leeds.ac.uk (A.M.); h.viswambharan@leeds.ac.uk (H.V.);; 3 Department of Nephrology, Leeds Teaching Hospitals NHS Trust, Leeds LS9 7TF, UK; andrew.mooney2@nhs.net; 4 Centre for Cardiovascular Science, The Queen’s Medical Research Institute, University of Edinburgh, Edinburgh EH16 4TJ, UK; laura.denby@ed.ac.uk

**Keywords:** beta blocker, cardiac remodelling, CKD, uremia

## Abstract

Heart failure and chronic kidney disease (CKD) share several mediators of cardiac pathological remodelling. Akin to heart failure, this remodelling sets in motion a vicious cycle of progressive pathological hypertrophy and myocardial dysfunction in CKD. Several decades of heart failure research have shown that beta blockade is a powerful tool in preventing cardiac remodelling and breaking this vicious cycle. This phenomenon remains hitherto untested in CKD. Therefore, we set out to test the hypothesis that beta blockade prevents cardiac pathological remodelling in experimental uremia. *Wistar* rats had subtotal nephrectomy or sham surgery and were followed up for 10 weeks. The animals were randomly allocated to the beta blocker metoprolol (10 mg/kg/day) or vehicle. In vivo and in vitro cardiac assessments were performed. Cardiac tissue was extracted, and protein expression was quantified using immunoblotting. Histological analyses were performed to quantify myocardial fibrosis. Beta blockade attenuated cardiac pathological remodelling in nephrectomised animals. The echocardiographic left ventricular mass and the heart weight to tibial length ratio were significantly lower in nephrectomised animals treated with metoprolol. Furthermore, beta blockade attenuated myocardial fibrosis associated with subtotal nephrectomy. In addition, the Ca^++^- calmodulin-dependent kinase II (CAMKII) pathway was shown to be activated in uremia and attenuated by beta blockade, offering a potential mechanism of action. In conclusion, beta blockade attenuated hypertrophic signalling pathways and ameliorated cardiac pathological remodelling in experimental uremia. The study provides a strong scientific rationale for repurposing beta blockers, a tried and tested treatment in heart failure, for the benefit of patients with CKD.

## 1. Introduction

**Figure 1:**
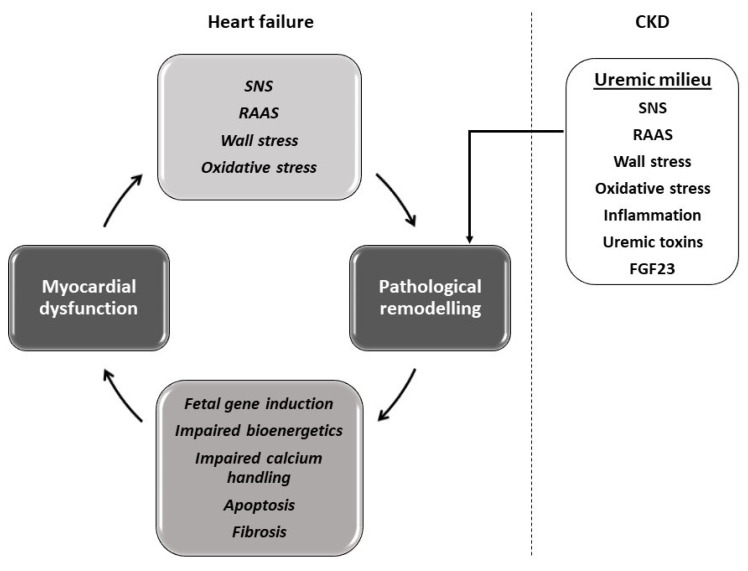
The central theme of several decades of heart failure research demonstrating a vicious cycle set in motion between pathological cardiac remodelling and myocardial dysfunction and the mechanisms and mediators of such pathology. The figure also shows the common mediators of pathological hypertrophy shared between heart failure and CKD. CKD: chronic kidney disease, SNS: sympathetic nervous system, RAAS: renin angiotensin aldosterone system, FGF23: fibroblast growth factor 23.

Left ventricular hypertrophy (LVH) is a ubiquitous finding in advanced chronic kidney disease (CKD) [1]. This hypertrophy is associated with a four-fold increase in the risk of heart failure (HF) in CKD [2], demonstrating that the mechanism involved is cardiac pathological remodelling wherein the remodelling leads to progressive myocardial dysfunction [3]. It is therefore not surprising that HF is the predominant cardiac disease of CKD and end-stage kidney disease (ESKD) [4]. This association between cardiac hypertrophy and HF is not unique to CKD. Indeed, the concept of a vicious cycle set in motion between cardiac hypertrophy and myocardial dysfunction leading to progressive heart failure is the central theme that emerged from half a century of HF research [5]. It is pertinent to note that HF and CKD share several potent risk factors of cardiac hypertrophy such as wall stress, renin angiotensin aldosterone system activation, sympathetic activation, oxidative stress and inflammation (Figure 1) [6,7,8,9,10]. Decades of HF research have also taught us that beta blockade is a powerful tool in breaking this vicious cycle by preventing progressive pathological remodelling and even inducing reverse remodelling [11,12,13]. This phenomenon remains hitherto untested in CKD despite the striking similarities between the pathogenesis of cardiac disease in CKD and HF. We therefore set out to test the hypothesis that beta blockade prevents cardiac pathological remodelling in experimental uraemia. We evaluated the cardiac effects of beta blockade, in vitro and in vivo, in a rodent model of CKD induced by subtotal nephrectomy.

In addition to studying the effects of beta blockade on cardiac structure and ultrastructure, we also evaluated the effect on the downstream signalling pathways of beta-adrenergic receptor (β-AR) to gain mechanistic insight. We assessed both the protein kinase A (PKA) pathway [14], the classic downstream effector of β-AR stimulation, and the Ca^++^-calmodulin-dependent kinase II (CAMKII) pathway, a novel downstream effector of β-AR and other hypertrophic stimuli that have been shown to play a major role in the genesis of cardiac pathological remodelling and subsequent HF [3,15].

## 2. Results

### 2.1. Biochemical Effects of Subtotal Nephrectomy

**Figure 2:**
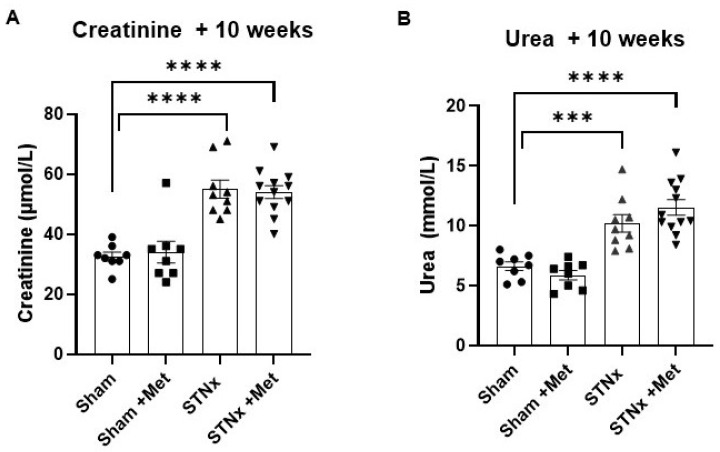
Subtotal nephrectomy increases serum creatinine and urea levels. Serum creatinine (µmol/L) (**A**) and urea (mmol/L) (**B**) at 10 weeks post-sham or -STNx ± metoprolol. (*** *p* < 0.001, **** *p* < 0.0001) (n = 7–12).

The subtotal nephrectomy (STNx) resulted in significant renal dysfunction, as shown in Figure 2 and Table 1. Compared to the control animals, the nephrectomised animals had a higher serum creatinine and serum urea levels at 10 weeks post-STNx. This demonstrates that STNx was successful in inducing uraemia, and metoprolol had no effect on kidney function as measured by serum creatinine and urea.

### 2.2. Assessment of Cardiac Structure

**Figure 3:**
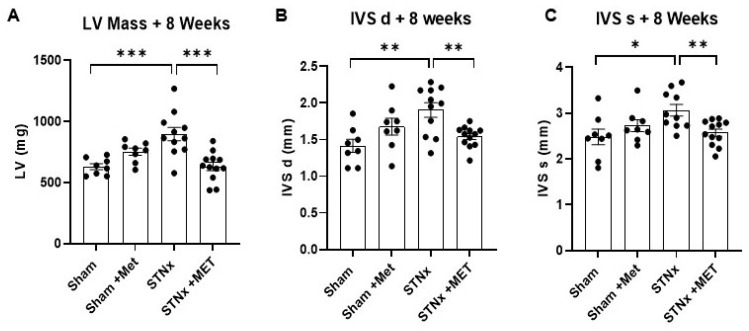
Metoprolol attenuates subtotal nephrectomy induced indices of cardiac mass. Echocardiographic assessment of left ventricular mass (**A**) and interventricular septal thickness in diastole (**B**) and systole (**C**), 8 weeks post-sham or -STNx ± metoprolol. (* *p* < 0.05, ** *p* < 0.01, *** *p* < 0.001) (n = 8–12).

The in vivo assessment of cardiac structure was performed using echocardiography at 8 weeks post-STNx. The results show that the animals that underwent STNx had significantly higher left ventricular mass than the control (sham) animals (Figure 3A). The LV mass was more than 40% higher in the STNx group compared to the sham group. This demonstrates that subtotal nephrectomy did indeed result in significant LV hypertrophy. In addition, subtotal nephrectomy also resulted in greater interventricular septal (IVS) thickness measured both during diastole and systole (Figure 3B,C). However, the STNx animals treated with metoprolol had lower LV mass and IVS thickness compared to the ones not treated with metoprolol. This demonstrates that the cardiac hypertrophic effect of uremia was significantly attenuated by betablockade therapy (Figure 3 and Table 2).

### 2.3. Assessment of Cardiac Function

**Figure 4:**
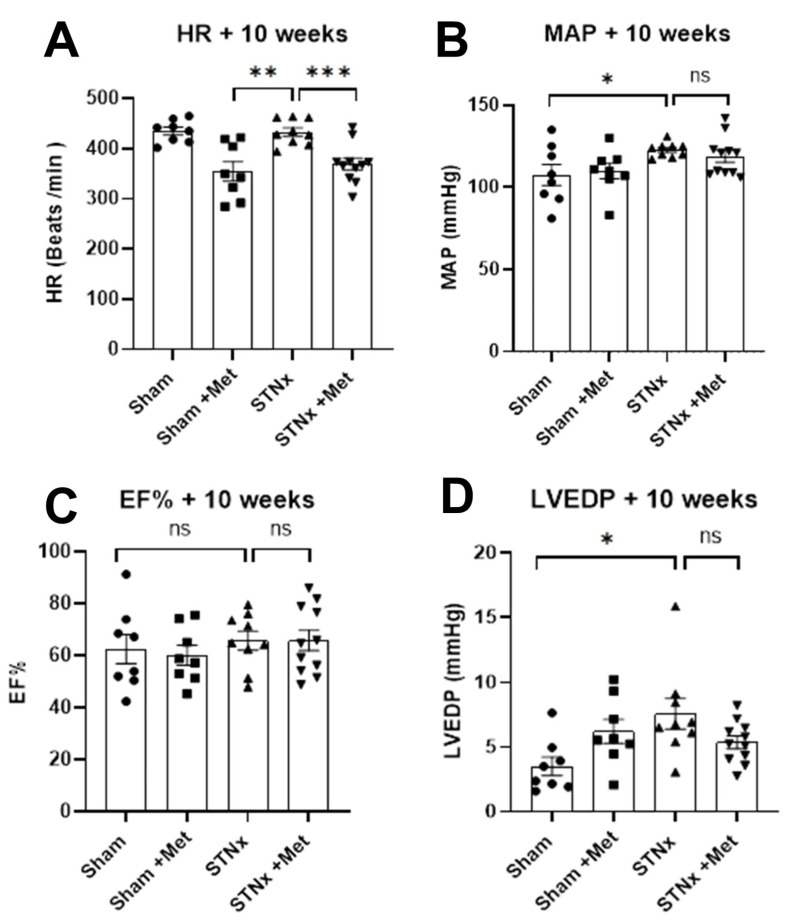
Effect of metoprolol on cardiac hemodynamics. Cardiac parameters as assessed by Millar pressure-volume conductance catheters. Heart rate (**A**), blood pressure (**B**), left ventricular ejection fraction (**C**) and end diastolic pressure (**D**) measured 10 weeks post-sham or -STNx ± metoprolol. (* *p* < 0.05, ** *p* < 0.01, *** *p* < 0.001) (n = 8–11).

The animals on metoprolol had a lower heart rate at 10 weeks compared to the ones not on metoprolol (Figure 4A and Table 2), demonstrating that effective beta blockade was achieved. At the same time, there was no difference in the mean arterial pressure between the nephrectomised animals that were on and not on metoprolol (Figure 4B). This ensures that the results of the study were not confounded by differential blood pressure control. Although there was no difference in the left ventricular ejection fraction (Figure 4C) between the study groups, invasive pressure volume loop analysis showed that the left ventricular end diastolic pressure was significantly elevated in nephrectomised animals, and there was a trend towards attenuation of this effect with metoprolol treatment (Figure 4D and Table 2).

### 2.4. Quantifying Cardiac Hypertrophy

**Figure 5:**
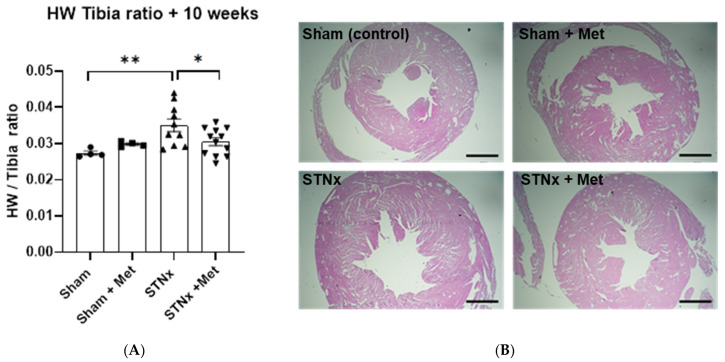
Metoprolol attenuates subtotal nephrectomy-induced left ventricular hypertrophy. Ventricular weight/tibia length ratio (cardiac weight index) from animals 10 weeks post-sham or -STNx ± metoprolol (**A**). Histological assessment of left ventricular mass 10 weeks post-sham or -STNx ± metoprolol (**B**). Hearts stained with H&E. (* *p* < 0.05, ** *p* < 0.01) (n = 4–12). Scale bars 200 µm.

In addition to the above-described in vivo measurements of cardiac dimensions, we also performed a more accurate quantification of cardiac hypertrophy by measuring heart weight normalised to tibia length after sacrificing the animals at 10 weeks. These measurements mirrored what was observed with echocardiogram. The animals that underwent STNx had significantly higher heart weight normalised to tibia length. The heart weight was greater by more than 27% in the nephrectomised animals compared to control (sham) animals. More importantly, the nephrectomised animals treated with metoprolol showed significantly lower heart weight compared to the ones not treated with metoprolol. This further reinforces the finding that beta blockade attenuates the cardiac hypertrophic effects of uremia (Figure 5A and Table 2). The H&E staining of the cardiac specimens from all four study groups shown in Figure 5B bears out this distinction visually.

### 2.5. Cardiac Fibrosis

**Figure 6:**
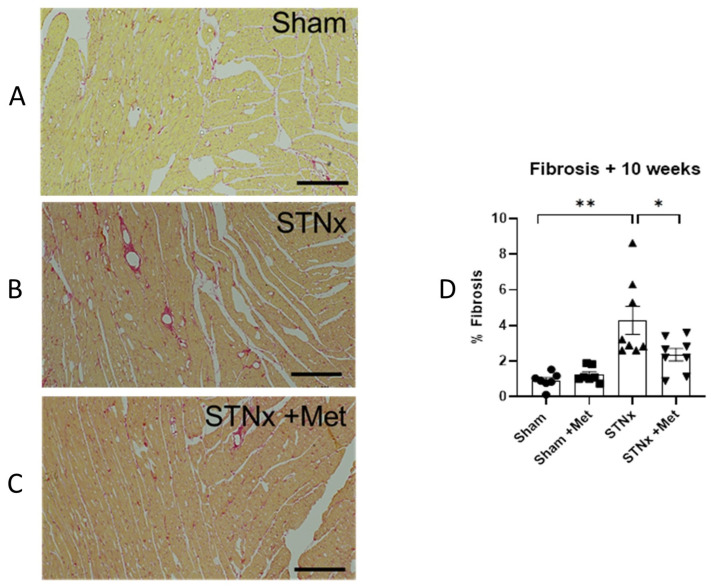
Metoprolol attenuates subtotal nephrectomy-induced cardiac fibrosis. Fibrosis is increased in ventricles of the heart 10 weeks post-nephrectomy, an effect attenuated with metoprolol. Representative histological images of 5µm sections of ventricle stained for collagen with picrosirius red in sham hearts (**A**) following STNx alone (**B**) and co-treatment with metoprolol (**C**). Summary of quantification of collagen staining in ventricles of rats 10 weeks post-sham or -STNx ± metoprolol (**D**). (* *p* < 0.05, ** *p* < 0.01) (n = 7–8). Scale bar 200 µm.

Qualitative and quantitative analyses of cardiac fibrosis were performed after staining the myocardium with picrosirius red staining at 10 weeks. Figure 6 shows the striking distinction in the extent of fibrosis between the study groups. Further quantitative analysis (Figure 6D) demonstrated that the nephrectomised animals showed significant interstitial expansion, with a %fibrosis of 4.30% compared to a %fibrosis of 0.91% in control (sham) animals (*p* < 0.05). More importantly, the nephrectomised animals treated with metoprolol had significantly lower interstitial expansion compared to the ones not on metoprolol, with a % fibrosis of 2.35% (*p* < 0.05). This demonstrates that betablockade therapy significantly attenuates the cardiac fibrotic effect of uremia. 

### 2.6. Mechanism of Action of Beta Blockade

To elucidate the mechanism of action of beta blckade in attenuating the cardiac hypertrophic effect of uremia, we performed immunobloting assays to quantify the protein expression of the downstream effectors of beta adrenoceptor signalling. We evaluated both the protein kinase A pathway, the well-known downstream effector of beta adrenoceptor signalling, and the novel calcium calmodulin kinase II (CaMKII) pathway.

#### 2.6.1. Inhibition of Calcium Calmodulin Kinase II (CaMKII) Pathway

**Figure 7:**
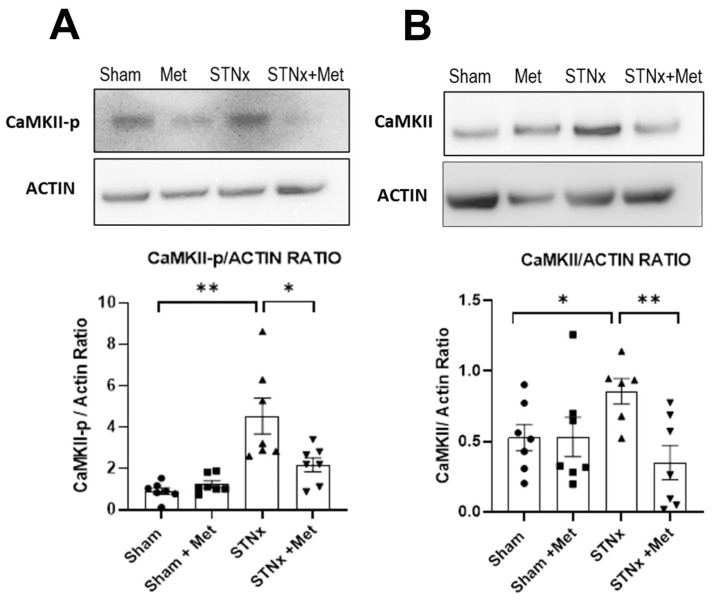
Metoprolol attenuates subtotal nephrectomy-induced CaMKII activation. Representative western blots of phosphorylated CaMKII (CaMKII-p) and beta-actin along with summary of CaMKII-p quantification (**A**) and representative western blot of total CAMKII and the corresponding summary of total CAMII quantification (**B**) in ventricles of rats 10 weeks post-sham or -STNx ± metoprolol. (* *p* < 0.05, ** *p* < 0.01) (n = 6–7).

To evaluate whether CAMKII activity was increased with STNx compared with control (sham), we assessed the protein expression of CAMKII and the phosphorylated CAMKII in the ventricular myocardium. Western blot analyis showed an increase in both total CaMKII and phosphorylated CaMKII following STNx. Thus, the study has shown for the first time that experimental uremia resulted in significant activation of CAMKII signalling in the myocardium. More importantly, the results also demonstrated that this signalling was attenuated by treatment with metoprolol (Figure 7). This demonstrates that the effect of betablockade in ameliorating cardiac hypertrophy and fibrosis in uremia shown above may be mediated by the attenuation of the CAMKII signal transduction pathway. 

#### 2.6.2. Inhibition of Protein Kinase A Pathway

**Figure 8:**
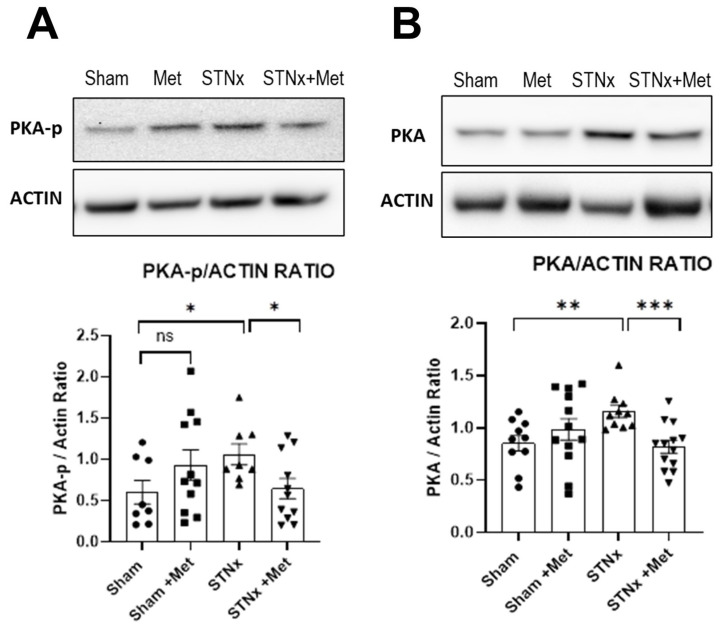
Metoprolol attenuates subtotal nephrectomy-induced PKA activation. Representative western blots of phosphorylated phosphorylate PKA (PKA-p) and beta-actin along with summary of PKA-p quantification (**A**) and representative western blot of total PKA and the corresponding summary of total PKA quantification (**B**) in ventricles of rats 10 weeks post-sham or -STNx ± metoprolol. (* *p* < 0.05, ** *p* < 0.01,*** *p* < 0.001) (n = 8–13).

The PKA signalling pathway, the classic beta adrenoceptor transduction pathway, was activated in the ventricles of hearts following nephrectomy, an effect attenuated by metoprolol. An increase in both phospho-PKA and total PKA following STNx was observed. Not surprisingly, this signalling was attenuated with co-treatment with metoprolol (Figure 8).

## 3. Discussion

The study demonstrated that beta blockade ameliorated cardiac pathological remodelling in experimental uremia. Both myocardial hypertrophy and interstitial fibrosis were attenuated. Beta blockade therapy was associated with almost 30% less LV mass and almost 50% less interstitial fibrosis in the uremic animals. The serum creatinine and urea were significantly elevated in the STNx group, demonstrating that STNx was successful in inducing uremia. The animals that received metoprolol had significantly lower heart rate, demonstrating that successful beta blockade was achieved. In the uremic animals, the average mean arterial pressure was comparable between the metoprolol group and the non-treatment group, suggesting that the beneficial effects of beta blockade shown here were over and above its blood pressure-lowering effect. 

The study also showed that STNx resulted in the activation of the cAMP-dependent protein kinase A (PKA) pathway and the Ca^++^-calmodulin-dependent kinase II (CAMKII) pathway. This strongly suggests that CAMKII is a potential signalling mechanism associated with cardiac pathological remodelling in uremia. More importantly, beta blockade attenuated these pathways, offering a potential mechanism of action for the beneficial effects of beta blockade.

The well-recognised cardiac hypertrophic stimuli such as wall stress, renin angiotensin aldosterone system activation, sympathetic activation, inflammation and oxidative stress are present in abundance in uremia. In addition, the uremic milieu also includes other hypertrophic stimuli such as fibroblast growth factor 23, uremic toxins, CKD mineral bone disorder and anaemia [6,7,8,9,10,16,17]. It is, therefore, not surprising that left ventricular hypertrophy (LVH) is the predominant cardiac structural abnormality of CKD, designated as uraemic cardiomyopathy (UCM), with >70% patients with CKD stages 4–5 showing left ventricular hypertrophy or concentric remodelling [1]. Furthermore, this hypertrophy causes a ~four-fold increase in the risk of incident HF in CKD [2]. An intervention that slows down the pathological remodelling could potentially prevent the development of HF and subsequent poor outcomes. 

Several decades of heart failure research have shown that the hypertrophic stimuli, both biochemical and mechanical, activate a complex network of intracellular signalling pathways in the cardiomyocytes, leading to pathological remodelling comprising of morphological, metabolic and electrical remodelling processes [3,18,19]. It has also been shown that molecular remodelling through reactivation of the fetal gene program plays a pivotal role [20,21]. These processes lead to cardiomyocte dysfunction, cardiomyocyte apoptosis and interstitial fibrosis, culminating in heart failure, which in turn causes further neurohormonal activation, setting in motion a vicious cycle (Figure 1) [5]. Heart failure research has also shown time and again that beta blockade has the greatest potential in breaking this vicious cycle and preventing or reversing the process of pathological remodelling [11,12,13,22]. This phenomenon remained hitherto untested in uremia, even though HF and CKD share a lot of these cardiac hypertrophic stimuli. The present study has demonstrated for the first time the potential for beta blockade in preventing cardiac pathological remodelling in uremia. 

Beta adrenergic receptor (βAR) downstream signalling includes the classic cAMP-dependent PKA pathway and the novel CAMKII pathway [23]. The PKA pathway regulates the excitation–contraction coupling. It has positive inotropic, chronotropic and lusitropic effects. Chronic PKA activation mediates cardiomyocyte apoptosis and necrosis [14]. In addition to the PKA pathway, there is growing interest in the role of the CAMKII signalling pathway. Chronic CAMKII activation is implicated in altered calcium homeostasis, proarrhythmic electrical remodelling, oxidative stress and apoptosis [24]. More importantly, the CAMKII pathway has emerged as the core mechanism that links the various hypertrophic stimuli to the activation of fetal gene program that leads to progressive myocyte hypertrophy and dysfunction [15]. Indeed, inhibition of the CAMKII signalling pathway has been shown to ameliorate the cardiac effects of hypertrophic stimuli, such as catecholamines, aldosterone and oxidative stress [25,26]. 

The present study showed that the CAMKII pathway is activated in uremia, suggesting that the signalling pathways involved in pathological remodelling in uremia are similar to that of a failing heart. Hence, not surprisingly, beta blockade demonstrated amelioration of pathological remodelling in uremia akin to its effect in failing heart. The uremic milieu comprises a myriad of cardiac hypertrophic stimuli, and it may not be feasible to remove or block all such stimuli. Instead, it may be that cardioprotection can best be achieved by mitigating the effects of such hypertrophic stimuli by targeting the common downstream pathways.

Observational studies and small clinical trials have shown the benefits of beta blocker therapy in patients with CKD or ESKD and pre-existing heart failure [27,28]. However, there are no published or ongoing randomised controlled trials assessing the benefits of beta blockers in the prevention of cardiac pathological remodelling in CKD. This study provides a strong rationale for translational research evaluating the benefits of beta blockade in patients with CKD. The study also opens new avenues of preclinical research in understanding the nature of UCM. A comprehensive study of the downstream targets of the CAMKII pathway, such as the fetal gene program, electrical remodelling and metabolic remodelling, and the effects of targeted inhibition of the CAMKII pathway in uremia would be worthwhile.

Although attempts were made in the 1980s to study the cardiac effects of various drugs including a beta blocker in experimental uremia, the short duration of the study (3 weeks) limited its ability to fully evaluate the cardiac effects of uremia and the benefits of potential therapies [29]. Nowadays, with much improved surgical techniques, the nephrectomised animals live significantly longer, allowing for the emergence of a uremic complication such as cardiomyopathy. 

The present study did not show any significant difference in the echocardiographic parameters of cardiac function (EF) between the study groups. Although the study was not aimed at demonstrating any early cardiac dysfunction, it is reassuring to note that betablockade therapy did not have an adverse effect on cardiac function. On the contrary, the left ventricular end diastolic pressure (LVEDP) was increased in the nephrectomised animals, and there was a trend towards reduction in LVEDP with beta blockade.

## 4. Materials and Methods

**Subtotal nephrectomy (STNx):** Chronic kidney disease was induced in rats using the subtotal nephrectomy model (STNx), which closely mimics human CKD and cardiorenal syndrome (Type 4). Experiments were performed in a manner we have previously described [30] and conducted in accordance with the Animals (Scientific Procedures) Act 1986, with the approval of the UK Home Office. Male *Wistar* rats (200–250 g) were housed 2–3 per cage in a temperature-controlled room set to a 12:12 h light–dark cycle with free access to food and water. Animals were randomised to receive sham or STNx surgery. Animal were anaesthetised, incisions made in the flank, and the right kidney located and nephrectomised. Similarly, the renal poles (approximately 2/3 renal mass) of the left kidney were then surgically removed. In each case, the adrenal gland was bluntly dissected away from the kidney to prevent adrenalectomy. For sham surgery, animals were prepared in a similar way: they had bilateral flank incisions and had both kidneys isolated and manipulated before wound closure. Following recovery from surgery, sham and STNx rats were randomly assigned to 2 groups to receive either beta adrenergic blocker (metoprolol 10 mg/kg/day) or vehicle for 10 weeks using Alzet Osmotic Pumps (models 2004/2006, Cupertino, CA, USA) implanted subcutaneously. Thereby, we have four groups to compare in this study: sham, mham+ metoprolol, STNx and STNx + metoprolol. We have successfully demonstrated that this dose of metoprolol is well tolerated in rats and within the human equivalent dose used clinically to treat left ventricular failure [31]. Animals were housed for 10 weeks. At 8 weeks, cardiac function was assessed by echocardiography, and at 10 weeks, by Millar catheter analysis. Following Millar catheter analysis, animals were sacrificed, and serum samples were taken for the measurement of creatinine and urea. Hearts were then harvested for protein analysis and histology (vide infra).

**Echocardiography**: Echocardiography was carried out 8 weeks post-STNx under general anaesthesia with 2% isoflurane in the manner we have described previously [32]. Echocardiography images were acquired on an ACUSON Sequoia (Universal Diagnostic Solutions, Vista, CA, USA) with a 15 MHz 15L8 transducer. All images were stored on optical media disks for subsequent offline analysis. M-mode recordings were taken in the parasternal short-axis view, allowing for recording of left ventricle anterior and posterior wall thickness and the internal diameter of the left ventricle in both systole and diastole. Right ventricle wall thickness was measured from M-mode recordings in the parasternal long axis view [32].

**Measurement of cardiac function and cardiac weight index:** Physiological measurements of cardiac function were obtained at the end of the experimental period by Millar conductance pressure–volume (PV) catheter analysis in a manner we have previously described [32,33]. Briefly, rats were anesthetised with isoflurane, and body temperature was maintained with a heating pad before inserting an ultra-miniature PV catheter SPR-869 (Millar Instruments, Houston Texas) into the left ventricle via the right carotid artery and ascending aorta. Data were collected via an MPVS-300 PV system (ADInstruments, Sydney, Australia), and PV loop analysis was performed with Chart 8 Pro software (ADInstruments). Heart rate (HR), LV end-systolic pressure (LVESP), LV end-diastolic pressure (LVEDP), the maximal slope of LV systolic pressure increment (dP/dtmax) and diastolic pressure decrement (dP/dtmin), LV end-systolic volume (LVESV), LV end-diastolic volume (LVEDV), stroke volume (SV), cardiac output (CO), ejection fraction (EF) and stroke work (SW) were computed and calculated.

On completion of haemodynamic measurements, blood was obtained from the left ventricle via cardiac puncture, and blood serum was assayed for creatinine and urea. The apex of each heart was sectioned, snap frozen and stored at −80 °C for protein analysis. The remainder of the heart was perfused-fixed in 4% paraformaldehyde via the left ventricle for histological analysis. Tibiae were collected, cleaned and measured. Cardiac weight index was calculated as the ratio of ventricular weight to tibia length [33].

**Immunoblotting:** Immunoblotting was performed in the manner we have previously described [33]. Briefly, ventricular tissue was lysed in extraction buffer containing (in mmol/L), 50 HEPES, 120 NaCl, 1 MgCl_2_, 1 CaCl_2_, 10 NaP_2_O_7_, 20 NaF, 1 EDTA, 0.2 PMSF, 2 sodium orthovanadate, 10% glycerol and 1% NP40, with 0.5 µg/mL leupeptin and 0.5 µg/mL aprotinin (Invitrogen Cell Extraction Buffer, FNN00 11). Tissue extracts were incubated for 30 min in an ice bath and centrifuged at 14,000 rpm for 15 min, before protein measurements were carried out by BCA assay kit (Pierce, Appleton, WI, USA), using the supernatant. For the analysis of total protein expression, equal amounts of cellular protein were resolved on SDS polyacrylamide gels (Invitrogen, Waltham, MA, USA) and transferred to polyvinylidene difluoride membranes. Membranes were then immunoblotted with appropriate antibodies: beta-actin (C4, sc-47778, Santa Cruz Biotechnology, Inc., Santa Cruz, CA, USA), CaMKIIδ (L-04, sc-100362 Santa Cruz Biotechnology, Inc.), p-CaMKII (T287, PA5-39731, Invitrogen), PKA C (4782, Cell Signaling Technology), and phospho PKA C (4781 Cell Signalling Technology, Danvers, MA, USA). Blots were incubated with appropriate peroxidise-conjugated secondary antibodies and developed with enhanced chemiluminescence imaging kit (Millipore, Burlington, MA, USA). Protein was quantified using densitometry (Syngene Gel Documentation System, Cambridge, UK) and normalised against beta-actin as loading control. All blots were repeated at least 6 times with independent samples, using identical experimental conditions. Representative blots were selected that were the nearest representation to the mean data presented that support the conclusions [33].

**Histology:** Three-micrometre-thick formalin-fixed paraffin-embedded sections of the heart were cut and deparaffinised prior to staining with either hematoxylin and eosin for morphological assessment or with picrosirius red for collagen deposition in accordance with manufacturer’s guidelines (ab150681, Abcam, Cambridge, UK). Slides were imaged using Nikon Bright-field ECLIPSE Ci-L with DS-L4 imaging. Collagen content was quantified from five fields of view (×10) from each of 6 sections using Image-Pro Premier 9.2.

**Statistics:** Data were analysed using an unpaired t-test with Welch’s correction and expressed as mean ± SEM. * *p* < 0.05, ** *p* < 0.01, *** *p* < 0.001 and, **** *p* < 0.0001. **N** = number of animals unless stated otherwise.

## 5. Conclusions

In conclusion, beta blockade attenuates hypertrophic signalling pathways and ameliorates cardiac pathological remodelling in experimental uremia. The study provides a strong scientific rationale for repurposing beta blockers, a tried and tested treatment in heart failure, for the benefit of patients with CKD.

### Disclosures

The authors have no relevant affiliations or financial involvement with any organisation or entity with a financial interest in or financial conflict with the subject matter or materials discussed in the manuscript. This includes employment, consultancies, honoraria, stock ownership or options, expert testimony, grants or patents received or pending or royalties. No writing assistance was utilised in the production of this manuscript. The manuscript represents an original work, was not invited for a special issue, is not under consideration elsewhere for publication in whole or in part and has not been published in another form or in another country, except in abstract format.

## Figures and Tables

**Table 1:** Biochemical Parameters at 10 Weeks.

	Sham	Sham + Metoprolol	STNx	STNx + Metoprolol
Creatinine (µmol/L)	32.05 ± 1.44	30.71 ± 1.81	55.00 ± 3.06	54.00 ± 2.20
Urea (mmol/L)	6.63 ± 0.36	5.88 ± 0.39	10.20 ± 0.72	11.53 ± 0.65

**Table 2:** Cardiac Parameters at 8 and 10 Weeks.

	Sham	Sham + Metoprolol	STNx	STNx + Metoprolol
HR (min^−1^)	434.74 ± 7.84	354.64 ± 19.45	432.72 ± 8.54	369.49 ± 11.80
MAP (mmHg)	107.50 ± 6.35	109.88 ± 4.72	122.78 ± 1.47	118.82 ± 3.60
Echo and PV loop parameters at 10 weeks				
EF (%)	62.54 ± 5.58	60.13 ± 3.81	65.74 ± 3.60	65.84 ± 3.95
LVEDP (mmHg)	3.51 ± 0.72	6.21 ± 0.93	7.57 ± 1.18	5.37 ± 0.49
Left ventricular mass at 8 weeks				
LV mass (mg)	629.81 ± 24.27	750.90 ± 29.45	896.37 ± 54.59	632.17 ± 34.04
Heart weight to tibial length ratio at 10 weeks				
HW:TL	0.0274 ± 0.0006	0.0299 ± 0.0004	0.0350 ± 0.0018	0.0304 ± 0.0011

STNx: subtotal nephrectomy, HR: heart rate, MAP: mean arterial pressure. EF: ejection fraction, LVEDP: left ventricular end diastolic pressure.

## Data Availability

Data are contained within this article.
